# Assessing the influence of sleep and sampling time on metabolites in oral fluid: implications for metabolomics studies

**DOI:** 10.1007/s11306-024-02158-3

**Published:** 2024-08-07

**Authors:** Michael Scholz, Andrea Eva Steuer, Akos Dobay, Hans-Peter Landolt, Thomas Kraemer

**Affiliations:** 1 Department of Forensic Pharmacology and Toxicology, Zurich Institute of Forensic Medicine, University of Zurich, Zurich, Switzerland; 2 Forensic Machine Learning Technology Center, University of Zurich, Zurich, Switzerland; 3 Institute of Pharmacology and Toxicology, University of Zurich, Zurich, Switzerland; 4 Sleep & Health Zurich, University of Zurich, Zurich, Switzerland

**Keywords:** Metabolomics, Sleep, Sleep deprivation, Oral fluid, Saliva, LC-MS

## Abstract

**Introduction:**

The human salivary metabolome is a rich source of information for metabolomics studies. Among other influences, individual differences in sleep-wake history and time of day may affect the metabolome.

**Objectives:**

We aimed to characterize the influence of a single night of sleep deprivation compared to sufficient sleep on the metabolites present in oral fluid and to assess the implications of sampling time points for the design of metabolomics studies.

**Methods:**

Oral fluid specimens of 13 healthy young males were obtained in Salivette^®^ devices at regular intervals in both a control condition (repeated 8-hour sleep) and a sleep deprivation condition (total sleep deprivation of 8 h, recovery sleep of 8 h) and their metabolic contents compared in a semi-targeted metabolomics approach.

**Results:**

Analysis of variance results showed factor ‘time’ (i.e., sampling time point) representing the major influencer (median 9.24%, range 3.02–42.91%), surpassing the intervention of sleep deprivation (median 1.81%, range 0.19–12.46%). In addition, we found about 10% of all metabolic features to have significantly changed in at least one time point after a night of sleep deprivation when compared to 8 h of sleep.

**Conclusion:**

The majority of significant alterations in metabolites’ abundances were found when sampled in the morning hours, which can lead to subsequent misinterpretations of experimental effects in metabolomics studies. Beyond applying a within-subject design with identical sample collection times, we highly recommend monitoring participants’ sleep-wake schedules prior to and during experiments, even if the study focus is not sleep-related (e.g., via actigraphy).

**Supplementary Information:**

The online version contains supplementary material available at 10.1007/s11306-024-02158-3.

## Introduction

The human metabolome is a rich source of information. To date, the Human Metabolome Database (HMDB) contains over 200’000 metabolite entries of different chemical classes (Wishart et al., 2022). Over the past decades, plenty of studies have exploited its potential, mostly for diagnostic, clinical, and forensic applications (Ashrafian et al., 2021; Johnson et al., 2016; Steuer et al., 2019; Szeremeta et al., 2021). Matrices frequently employed in metabolomics research encompass blood, urine, and increasingly, oral fluid. The latter, often synonymously but inaccurately referred to as saliva, is, by definition, the entirety of liquid present in the oral cavity. Its composition is made of the secreted products of salivary glands (i.e., saliva), microbiota, lining cells, and extrinsic substances – sometimes mixed with other fluids such as bronchial or nasal secretions, or blood in case of oral bleedings (Bellagambi et al., 2020). Time-of-day variations in flow rates and the sampling location, due to the unequal spread of different gland types in the oral cavity, have been shown to affect its composition (Ciurli et al., 2024). The simple, cheap, non-invasive, and repeatable sampling nature in combination with relevant biological information has made oral fluid an appealing matrix of choice for metabolomics studies, regardless of the study topic (Gardner et al., 2020).

Analytical setups in metabolomics typically involve the use of separation techniques (e.g., liquid chromatography (LC), gas chromatography, capillary electrophoresis, among others) in conjunction with mass or structural analyzers that utilize mass spectrometry (MS) or nuclear magnetic resonance spectroscopy for the instrumental analysis of biological samples. However, various instrumental configurations can be employed to meet particular analytical requirements. Currently, no comprehensive approach is available to analyze the complete set of metabolites present in a biological system (Tebani et al., 2018). Recent advancements in mass spectrometry technology have led to significant enhancements in sensitivity, enabling the measurement of a broader range of analytes, including those present in very low abundance (Li et al., 2021). Thus, even small differences between study groups can be detected and characterized. However, the potential advantages of increased detection rates must be weighed against the increased possibility of identifying spurious associations resulting from confounding factors in research designs (Collins et al., 2021). This phenomenon contributes to the lack of successful translation of numerous biomarkers into clinical assays (Crutchfield et al., 2016). Among the generally known influencers of metabolomics studies such as age, sex, body mass index, diet, health and medication use, smoking status, and physical activity, it has been clearly shown that circadian rhythm and time of day impact the metabolome (Gu et al., 2019; Kim et al., 2014; Slupsky et al., 2007; Sugimoto et al., 2013; Tolstikov et al., 2020; Walsh et al., 2006). Although researchers usually put good effort into excluding, controlling, or minimizing the aforementioned confounding variables when designing a metabolomics study, they often forget or neglect the influence of individuals’ sleep-wake histories – especially if the study matter is not related to sleep at all.

Sleep is a vitally important process in the animal kingdom. Humans spend about one-third of their lifetime sleeping, with vast differences in daily sleep need during different stages of life (Chaput et al., 2018). It is understood that sleep is an active restorative process with a complex but coordinated cascade of metabolic activities. Chronic disruptions in these mechanisms due to irregular or changing sleep-wake schedules (e.g., in shiftwork) have been associated with metabolic diseases such as diabetes and obesity (Knutsson & Kempe, 2014; Zhang et al., 2020). Furthermore, acute disruption of sleep patterns (e.g., a single night of total sleep deprivation) influences brain function, cognitive performance, and immune function (Garbarino et al., 2021; Hudson et al., 2020). It is, therefore, not surprising that a night of little or no sleep affects metabolite levels in biological matrices (Davies et al., 2014).

Sleep-related metabolomics primarily centers on rhythmic alterations, as it is based on a periodic phenomenon. Circadian rhythms follow a continuous, nearly 24-hour cycle driven by the suprachiasmatic nucleus and remain periodic without the influence of external time cues (i.e., they persist under constant routine conditions). Diurnal rhythms occur under stable real-life conditions (i.e., entrained conditions) but disappear without the influence of rhythmic exogenous factors (e.g., light conditions, sleep/wake cycles, or fasting/feeding cycles). Under real-life conditions, both of these rhythms contribute to time-of-day variation in metabolite profiles (Franken & Dijk, 2023). As shown in a comprehensive review of publications, only a few studies have dealt with time-of-day variation of salivary metabolites (Hancox et al., 2021). Furthermore, there is virtually no knowledge about the effect of sleep (or the loss of sleep) on metabolite levels in oral fluid. Since sleep deprivation and prolonged wakefulness have been shown to cause distinct metabolic changes in plasma (Davies et al., 2014), we hypothesized that this is also the case for oral fluid.

Thus, the aim of this study was to characterize the influence of a single night of sleep deprivation compared to sufficient sleep on the metabolites present in oral fluid and to assess its possible implications for the design of metabolomics studies in an era of detection of low abundant analytes.

## Materials and methods

### Chemicals, reagents, and materials

Salivette^®^ sampling devices were obtained from Sarstedt (Sevelen, Switzerland). Methanol and acetonitrile (Optima^®^ LC–MS grade) and ammonium acetate were purchased from Fisher Scientific (Basel, Switzerland), water (LC–MS grade) from VWR (Dietikon, Switzerland). Formic acid (Ultra Liquid Chromatography-MS grade) was obtained from Biosolve (via Chemie Brunschwig AG, Basel, Switzerland). All heavy-labeled and deuterated internal standards (ISTD) arginine-13C_6_, deoxycholic acid-*d*_4_, phenylalanine-*d*_1_, and proline-15 N were purchased from Cambridge Isotope Laboratories, Inc. (Andover, MA, USA) and delivered by ReseaChem GmbH (Burgdorf, Switzerland) or Sigma-Aldrich (Buchs, Switzerland). Compound reference standards of acetylcarnitine (C2), adenine, adenosine, arginine, butyrylcarnitine (C4), cortisol, cortisone, creatinine, glutaric acid, glycine, glycocholic acid, hippuric acid, inosine, carnitine (C0), leucine, lysine, methylmalonic acid, ornithine, phenylalanine, proline, propionylcarnitine (C3), raffinose, riboflavin, taurine, tryptophan, tyrosine, uracil, uric acid, uridine, and valine, as well as ammonium formate and acetic acid (LC-MS quality) were purchased from Sigma-Aldrich (Buchs, Switzerland). Eppendorf tubes (1.5 mL) were delivered by Eppendorf SE (Hamburg, Germany), and conic HPLC vials were supplied from infochroma ag (Goldau, Switzerland).

### Study

**Fig. 1:**
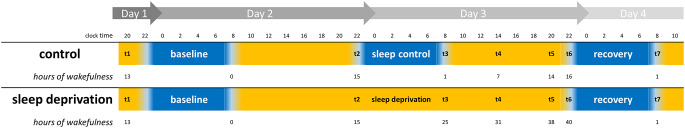
Different sleep regime timelines and amount of hours of sustained wakefulness of both study interventions (control, sleep deprivation) during the experimental phase in the sleep laboratory. Orange bars indicate time awake; blue bars indicate time asleep. t1-t7 represent oral fluid sampling time points

During a randomized, crossover, controlled sleep study, oral fluid specimens of healthy young male participants were obtained at regular intervals (day 1: 08:30 pm, day 2: 10:55 pm, day 3: 08:10 am, 01:55 pm, 09:00 pm, 10:55 pm, day 4: 08:10 am). The study protocol is described in detail elsewhere (Hefti et al., 2013). In brief, the study participants were selected based on strict inclusion criteria related to their sleep quality and psychological well-being. Additionally, they refrained from using medication or drugs during the study period and from caffeine and alcohol three days prior to each experimental block, controlled through measuring caffeine levels in saliva and breath alcohol tests. All subjects were served meals at the same time points. They strictly followed a sleep-wake schedule of 8 h of sleep and 16 h of wakefulness in the pre-experiment phase, which was confirmed through wrist actigraphy and sleep logs. The study involved two distinct experimental sessions, namely the control and sleep deprivation conditions, wherein the sole variable was the schedule of sleep and wakefulness (see Fig. 1). During the control condition, participants adhered to their 16 h/8 h wake/sleep protocol (time in bed from 11:30 pm to 07:30 am) for a further three nights (baseline, sleep control, and recovery night) in the sleep laboratory. Under the sleep deprivation condition, the initial night served as the baseline and was succeeded by 40 consecutive hours of wakefulness. Throughout this period, the participants were closely monitored by the research team. In both conditions, the recovery night provided a total of 8 h of sleep opportunity. Oral fluid samples were collected on sterile cotton swabs in Salivette^®^ devices (Sarstedt AG, Sevelen, Switzerland) and stored immediately at -20 °C until extraction in summer 2021 without intermittent freeze-thaw cycles. Out of the initial sample sets consisting of 22 people, the sets of 13 people (control and sleep deprivation for each participant) were available for this study. For the demographic characteristics of the participants, see Supplementary Information 1.

### Sample preparation

The sample preparation followed a simple and established approach: debris/protein removal and “dilute and shoot.” After thawing, Salivette^®^ devices were centrifuged for 2 min at 1’000 *g* according to the manufacturer’s protocol. From each sample, 200 µL of supernatant oral fluid were pipetted to Eppendorf tubes, and 600 µL of cold acetonitrile (-20 °C) were added. After vortexing for 10 s, the tubes were shaken in ThermoMixer (Vaudaux-Eppendorf AG, Schönenbuch, Switzerland) at 1400 rpm for 10 min and incubated overnight at -20 °C. The following day, tubes were thawed, vortex mixed (15 s), and centrifuged (5 min at 20’000 *g*). 200 µL of protein-free supernatant was transferred to conic glass vials, and 20 µL aqueous ISTD solution (arginine-13C_6_ (1.5 mM), deoxycholic acid-*d*_4_ (0.009 mM), phenylalanine-*d*_1_ (1.5 mM), and proline-15 N (3.5 mM) was added to monitor chromatographic performance consistency over the measurement period. A process blank sample (using LC-MS grade water instead of oral fluid in the Salivette^®^ device, i.e., a blind sample) was prepared in the same manner to correct for false positive results, and additionally, pooled quality control (QC) samples were prepared by mixing equal parts of each final filtrate. Lastly, a dilution series of QC pool samples (100%, 50%, 20%, 10%) was made to assess linearity of metabolic features during data cleaning procedures (Broadhurst et al., 2018).

### Instrumental analysis

LC-MS analysis was performed as described in detail elsewhere (Boxler et al., 2019). In short, a Thermo Fischer Ultimate 3000 UHPLC system (Thermo Fischer Scientific, San Jose, CA, USA) was equipped with two different columns, separately. For reversed-phase (RP) chromatography, a Waters (Baden-Daettwil, Switzerland) XSelect HSST RP‐C18 column (150 mm x 2.1 mm i.d., 2.5 μm particle size), and for hydrophilic interaction chromatography (HILIC), a Merck (Darmstadt, Germany) SeQuant ZIC HILIC column (150 mm x 2.1 mm i.d., 3.5 μm particle size) was used. The LC system was coupled to a high‐resolution (HR) quadrupole-time-of-flight (QTOF) instrument system (TripleTOF 6600, Sciex, Concord, Ontario, Canada), equipped with an electro spray ionization (ESI) source running in both positive and negative ionization modes. Thus, four different LC-MS modes were tested: RP+, RP-, HILIC+, and HILIC-. For detailed gradient profiles and instrument settings, see Supplementary Information 2. Data-dependent acquisition (DDA, top 5) mode was used for generation of HR (tandem) mass spectra (MS1, MS2), which was controlled by Analyst TF software 1.7 (Sciex). After every 5 samples, the mass spectrometer was recalibrated using the manufacturer’s mass calibration solution.

After system suitability assessment of model substances (arginine, cortisol, cortisone, creatinine, glycocholic acid, hippuric acid, leucine, raffinose, riboflavin, and tryptophan) as described by Steuer et al. (Steuer et al., 2017) and system equilibration by injection of ten QC pool samples, the prepared study samples were analyzed batch-wise in randomized order. To monitor system stability and for further signal adjustments, QC pool samples were reinjected every 5 samples. Dilution series of QC pool samples were measured at the beginning and end of the sample sequence.

### Data analysis

For targeted analysis of specific metabolites, MultiQuant 2.1 software (Sciex) was used for peak integration of raw MS data. The identification of these metabolites was confirmed by an in-house database built upon certified reference standards. In case missing values were present, they were substituted with one-fifth of the lowest measured peak area of the corresponding metabolite, following the 1/5 limit of detection (LOD) method. Raw peak areas were then log-transformed (base 10) to meet requirements of subsequent parametric statistical models, which were applied using GraphPad Prism 10 (GraphPad Software, San Diego, CA, USA). Z-score normalization (i.e., auto-scaling) was applied to overcome expected individual differences in absolute metabolite abundance scales. That is, for each molecular feature (MF), the abundance value was subtracted by the subject’s mean value and divided by its respective standard deviation. Thus, the resulting values (z-scores or z-distribution) have a mean of zero and a standard deviation of one, which allows for comparison of rhythmic metabolites irrespective of their amplitude.

For untargeted metabolomics analysis, raw MS data processing (alignment, deconvolution, peak picking) was performed with Progenesis Qi software (version 2.4.69, Waters Corp., Milford, USA). The reference for RT alignment was a pooled QC sample, and peak picking was executed utilizing the following parameters: automatic sensitivity, value: 3, no minimum peak width, retention time limit > 0.5 min (void volume), ion species: [M + H]^+^, [M + 2H]^2+^, [M + H-H_2_O]^+^, [M + NH_4_]^+^, [M + Na]^+^, [M + 2Na]^2+^, [M-H]^−^, [M-2H]^−^, [M-H_2_O-H]^−^, [M + Na-2H]^−^, [M + HCOOH-H]^−^. The resulting table of MFs’ raw abundances was exported, and subsequent data manipulation was performed in Python environment (Spyder version 5.1.5, Python version 3.9.12) using nPYc-Toolbox module (version 1.2.6) (Sands et al., 2019). Firstly, batch and run-order correction was applied with reference to QC pool samples (adapted LOWESS approach proposed by Dunn et al. (Dunn et al., 2011), parameter batch_correction_window = 8). Secondly, feature filtering was performed based on the following quality criteria (Broadhurst et al., 2018; Lewis et al., 2016): MF removed if present in process blank sample, or relative standard deviation (RSD) in QC pool samples > 25%, or correlation to dilution r^2^ < 0.7 in linear regression, or dispersion ratio (“D-ratio”) > 50%. In addition, features and individuals with more than 30% missing or zero values were excluded from subsequent statistical analyses. Missing or zero values of remaining features were replaced by one fifth of the lowest value detected (1/5 LOD method). In total, 29’957 values had to be replaced (7.1% of all values). The resulting feature table was log-transformed (base 10) for subsequent parametric testing. Applied feature selection, transformation, statistical analyses, and visualizations were conducted using MetaboAnalyst 5.0 (Pang et al., 2021), GraphPad Prism 10, and seaborn Python package (version 0.12.2).

### Data evaluation

**Fig. 2:**
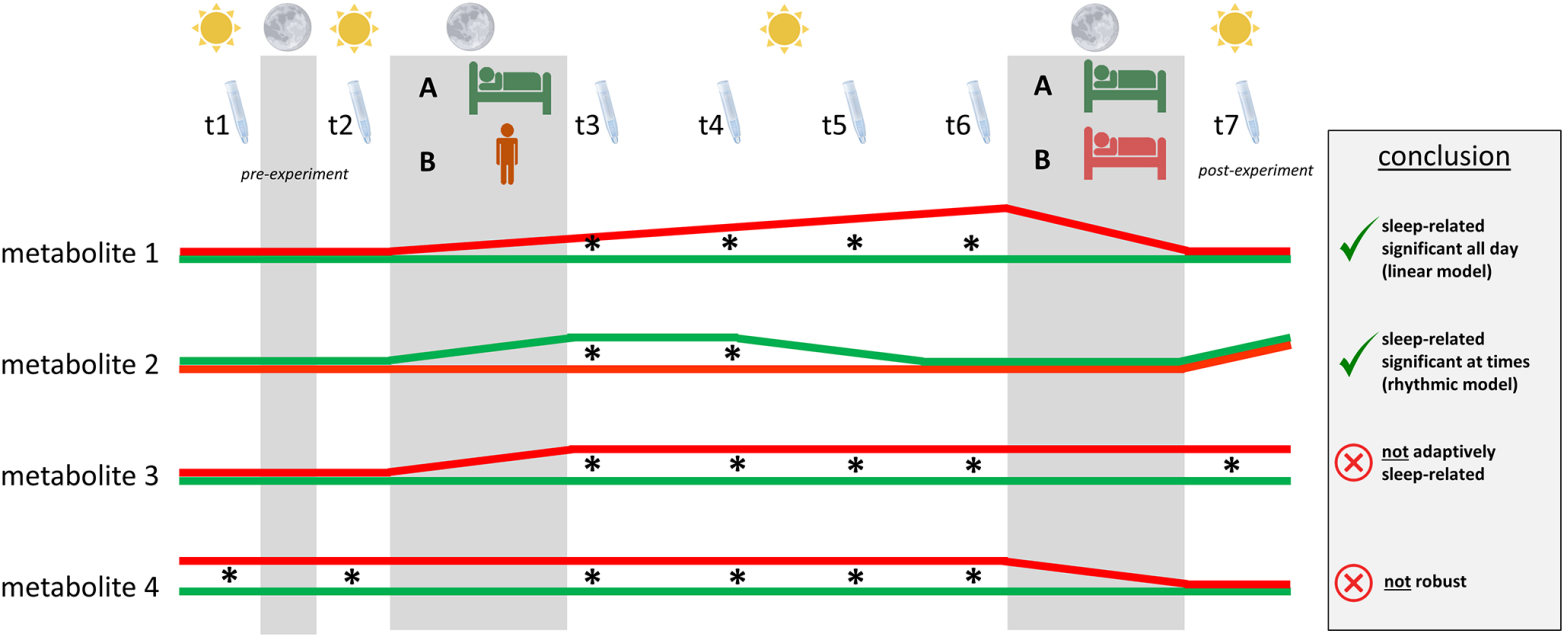
Theoretical profiles of fictitious metabolites. Sun icon indicates biological day, moon icon and gray fields indicate biological nighttime. t1-t7 and Salivette^®^ devices highlight necessary sampling time points of which t1 and t2 should be before the experimental night, and t7 after recovery night. Group A represents the control condition that offers sleep in both the second (experimental night) and the third biological night (recovery). Group B represents the sleep deprivation condition, which is kept awake during the experimental night but allows recovery sleep in the following night. Theoretically expected levels of four different metabolite types are portrayed in either green or red lines for the different conditions A and B, respectively. Asterisks indicate sampling time points when a statistical analysis results in a significant difference between the two metabolite levels. Metabolite 1 mimics a sleep-related metabolite following a linear model as the changes in metabolite levels occur during experimental night, increase linearly during the following daytime, and recede after recovery night. Likewise, metabolite 2 is sleep-related, as changes occur during the experimental night but can only be detected at sampling time points t3 and t4. This holds true for metabolites that are influenced by daily rhythm patterns under natural conditions (i.e., rhythmic model). Levels of metabolite 3 do not align after recovery night and can therefore not be related to adaptive sleep-wake regulation. Metabolite 4 shows significant differences between the two conditions even before the experiment starts and can thus not be reliably used for such studies

If metabolic changes are driven by rhythmic processes, they may only occur in one or two time points after the sleep deprivation/sleep control night, whereas linear processes would lead to significant differences in all four time points after sleep deprivation. These repercussions for data interpretation are schematically illustrated in Fig. 2, where expected levels of four different metabolite types are portrayed (referring to sampling time points t3, t4, t5, and t6). Very importantly, the sampling time point after the recovery night (t7) serves as a proof-of-concept control. The underlying rationale is that metabolic alterations are only due to prolonged wakefulness if they recede to baseline after recovery sleep (i.e., adaptive sleep-wake regulation). Therefore, if metabolite abundances measured after recovery night are of significant difference between the sleep deprivation condition and the control condition, they cannot be associated with adaptive sleep-wake regulation and are hence to be excluded from further investigation. Likewise, no statistical significance is expected between metabolites of both study interventions in t1 and t2 samples, which adds further robustness to the results.

### Analysis 1: influence of time and sleep on total variance (exemplified for targeted metabolites)

In order to measure the effects of the sampling time points and sleep on the overall variance of the targeted metabolites, we conducted repeated measures two-way analyses of variance (ANOVA). This statistical approach uses a general linear model and multiple measurements to examine the impact of two categorical independent variables (factors) on a continuous dependent variable. The output provides information about whether there are significant main effects for each factor (here: the sampling time points and the sleep deprivation intervention, labeled as time and sleep), and whether there is a significant interaction effect between these two factors. Additionally, the subject effect (i.e., inter-individual variation) is calculated by percentage of total variation. The analysis was run on data gathered between sampling time points t3 and t6 (when the effects of sleep deprivation are to be observed), and to account for the short time intervals between measurements, the Greenhouse-Geisser correction was employed, leading to more conservative (i.e., larger) p-values and to prevent the inflation of Type I error rates. Applied models for each metabolite were checked for residual distribution, homoscedasticity, and normality. The strong influence of interindividual differences on metabolite levels poses a substantial challenge in the identification of reliable biomarkers. To control or mitigate this effect, metabolomics studies usually apply a within-subject design and normalize the respective datasets. In our case, a subject-wise z-score normalization was applied, and the analyses were rerun.

### Analysis 2: Influence of sleep deprivation on metabolites’ abundances

The transformed dataset was analyzed by multiple paired t-tests for each sampling time point between the sleep deprivation and the control condition to identify alterations in abundances of metabolites. In order to address the issue of multiple testing, the significance level alpha was reduced to 0.01 for the untargeted metabolite analysis. Additionally, the same stringent dropout criteria were applied to time points t1, t2, and t7 as mentioned previously. This adjustment allows for a false positive rate of 0.97% (calculated as 0.01 multiplied by 0.99 three times) for a molecular feature to be considered significantly altered in a single time point between t3 and t6.

### Metabolite identification

Various databases (HMDB (Wishart et al., 2022), METLIN (Guijas et al., 2018), and the National Institute of Standards and Technology (NIST) were queried via Progenesis Qi for features of interest at both the MS1 and MS2 levels. In addition, SIRIUS software (version 5.8.3 (Dührkop et al., 2019)) was used for molecular formula deduction and its tools CANOPUS (Dührkop et al., 2021) and CSI: FingerID (Dührkop et al., 2015) for compound class prediction and structure annotation. If available, the identity confirmation was achieved through the correlation of library search outcomes, precise precursor masses and fragment ions, and retention time with certified reference standards. Based on the Metabolomics Standards Initiative (MSI) convention (Sumner et al., 2007), tentative identification confidences were categorized into four levels, ranging from 1 (highest confidence) to 4 (unknown compound).

## Results

We applied a semi-targeted approach: In a first analysis, we focused on 25 metabolites that are often reported due to either their physiological relevance or their presence in standard reference kits (i.e., targeted analysis). These metabolites are summarized in Table 1. In a second analysis, the range was broadened to also include all tentatively identified or unknown features (i.e., untargeted or global metabolomics analysis).

**Table 1:** Results for repeated measures two-way ANOVA

	HMDB ID	LC-MS setup		non-normalized dataset			z-normalized dataset			*P* value		
				% of total variation			% of total variation					
		column	polarity	‘time’	‘sleep’	subject	‘time’	‘sleep’	subject	‘time’	‘sleep’	‘time’ x ‘sleep’
Adenine	HMDB0000034	HILIC	positive	1.94	5.91	50.56	3.71	12.46	4.70	0.159	0.016 (*)	0.024 (*)
Adenosine	HMDB0000050	RP	positive	4.95	0.34	31.39	7.22	0.93	2.04	0.105	0.390	0.112
Arginine	HMDB0000517	HILIC	positive	8.56	0.62	43.87	13.69	1.88	7.19	0.006 (**)	0.294	0.027 (*)
C0 (Carnitine)	HMDB0000062	HILIC	positive	1.76	0.63	53.87	3.02	1.88	6.13	0.390	0.269	0.782
C2 (Acetylcarnitine)	HMDB0000201	HILIC	positive	1.33	0.50	55.60	5.02	1.26	5.10	0.177	0.343	0.477
C3 (Propionylcarnitine)	HMDB0000824	HILIC	positive	1.52	1.22	45.47	3.62	3.30	6.34	0.272	0.143	0.373
C4 (Butyrylcarnitine)	HMDB0002013	HILIC	positive	4.60	4.32	52.41	8.48	11.10	5.90	0.020 (*)	0.004 (**)	0.141
Cortisol	HMDB0000063	RP	negative	5.10	0.32	5.67	5.06	0.37	7.18	0.201	0.643	0.012 (*)
Cortisone	HMDB0002802	RP	positive	35.06	0.25	7.23	42.91	< 0.01	2.83	< 0.0001 (****)	0.979	0.673
Creatinine	HMDB0000562	HILIC	positive	1.11	0.46	63.21	4.48	0.95	10.34	0.132	0.429	0.216
Glutaric acid	HMDB0000661	HILIC	negative	7.22	0.19	48.03	12.93	1.66	4.49	0.009 (**)	0.310	0.701
Glycine	HMDB0000123	HILIC	negative	9.55	2.48	46.66	18.59	5.19	5.97	< 0.0001 (****)	0.092	0.380
Inosine	HMDB0000195	HILIC	negative	11.96	0.02	26.67	13.94	0.19	4.31	0.007 (**)	0.764	0.327
Leucine	HMDB0000687	HILIC	negative	4.16	1.14	39.22	7.54	2.68	7.53	0.037 (*)	0.229	0.877
Lysine	HMDB0000182	HILIC	negative	7.52	4.22	47.52	12.93	8.94	6.99	0.003 (**)	0.026 (*)	0.336
Methylmalonic acid	HMDB0000202	HILIC	negative	10.76	0.03	27.44	19.70	0.55	4.80	0.003 (**)	0.525	0.053
Ornithine	HMDB0000214	HILIC	negative	7.22	0.28	44.72	12.74	1.50	6.50	0.010 (**)	0.344	0.124
Phenylalanine	HMDB0000159	HILIC	negative	3.92	1.27	64.85	12.82	2.83	4.27	0.008 (**)	0.183	0.647
Proline	HMDB0000162	HILIC	positive	3.47	3.28	46.41	6.62	7.61	5.49	0.086	0.036 (*)	0.116
Taurine	HMDB0000251	HILIC	negative	3.20	0.10	52.68	4.80	0.76	7.31	0.226	0.358	0.431
Tyrosine	HMDB0000158	HILIC	negative	10.62	3.72	44.82	18.64	7.46	6.70	0.001 (***)	0.013 (*)	0.091
Uracil	HMDB0000300	HILIC	negative	7.17	1.24	34.33	9.99	1.81	8.97	0.014 (*)	0.409	0.802
Uric acid	HMDB0000289	HILIC	negative	1.47	0.99	37.97	4.19	1.36	3.83	0.236	0.311	0.006 (**)
Uridine	HMDB0000296	HILIC	negative	8.90	0.41	30.70	11.44	0.27	5.29	0.022 (*)	0.666	0.421
Valine	HMDB0000883	HILIC	positive	7.93	1.19	12.01	10.88	1.09	3.59	0.035 (*)	0.322	0.108
			MEDIAN	5.10	0.63	44.82	9.24	1.81	5.94			
			MIN	1.11	0.02	5.67	3.02	< 0.01	2.04			
			MAX	35.06	5.91	64.85	42.91	12.46	10.34			

Asterisks indicate p values: *p* < 0.05 (*); *p* < 0.01 (**); *p* < 0.001 (***); *p* < 0.0001 (****)

### Results of quality controls

System suitability was verified by assessment of peak areas of model substances. The mean relative standard deviations were 6%, 13%, 10%, and 8% for RP+, RP-, HILIC+, and HILIC- setup, respectively. Chromatographic performance consistency was confirmed by monitoring retention time shifts of ISTD across all batches. The differences between minimal and maximal retention times were on average 0.07, 0.18, 0.14, and 0.07 min for arginine-13C_6_, deoxycholic acid-*d*_4_, phenylalanine-*d*_1_, and proline-15 N, respectively. For single results, please refer to Supplementary Information 3.

### Analysis 1: Influence of factors ‘time’ and ‘sleep’ on total variance (exemplified for targeted metabolites)

The results of the analyses are presented in Table 1. In the non-normalized dataset, the subject effect (median 44.82% of total variation, range 5.67–64.85%) outshined both the factors ‘time’ (median 5.10%, range 1.11–35.06%) and ‘sleep’ (median 0.63%, range 0.02–5.91%). The output for the z-normalized dataset suggests mitigation of the inter-individual variation, as the subject effect decreased substantially (median 5.94% of total variation, range 2.04–10.34%). It became apparent that ‘time’ (i.e., sampling time point) represents the major influencer (median 9.24%, range 3.02–42.91%), surpassing the intervention of sleep deprivation (median 1.81%, range 0.19–12.46%). In fact, 15 out of the 25 investigated metabolites were significantly impacted by ‘time’, whereas 5 out of 25 were significantly impacted by sleep deprivation. The interaction between these two factors was significant for 4 out of 25 metabolites (refer to Table 1). The main effect of ‘time’ was predominantly observed for the rhythmic metabolites cortisone and glycine (*p* < 0.0001, see Fig. 4d and i).

### Analysis 2: Influence of sleep deprivation on metabolites’ abundances

**Fig. 3:**
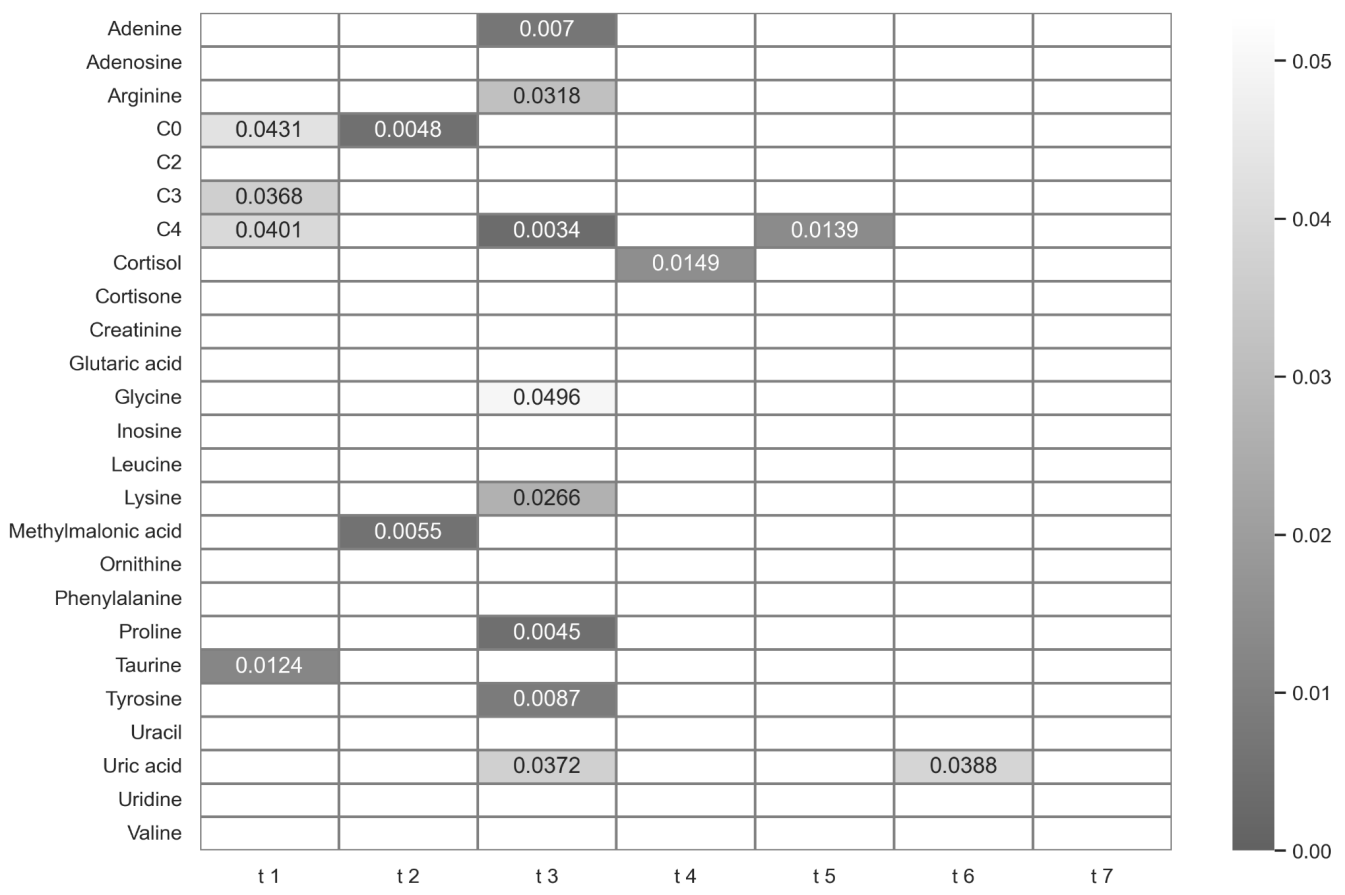
Heat map of p-values of paired t-tests for targeted metabolites between the sleep deprivation and the control condition across different sampling time points. Values below 0.05 are colored in grey shades and annotated. Values higher than 0.05 are represented in white boxes

In the field of biomarker research, researchers aim to identify significant differences in metabolite abundances across various experimental conditions. The results of the statistical testing for the targeted metabolites between time points are displayed in a heat map in Fig. 3. As outlined above, changes are only sleep-related if they occur at sampling time points t3-t6. Additionally, an insignificant outcome is sought for analytical robustness at t1, t2, and t7, respectively.

**Fig. 4:**
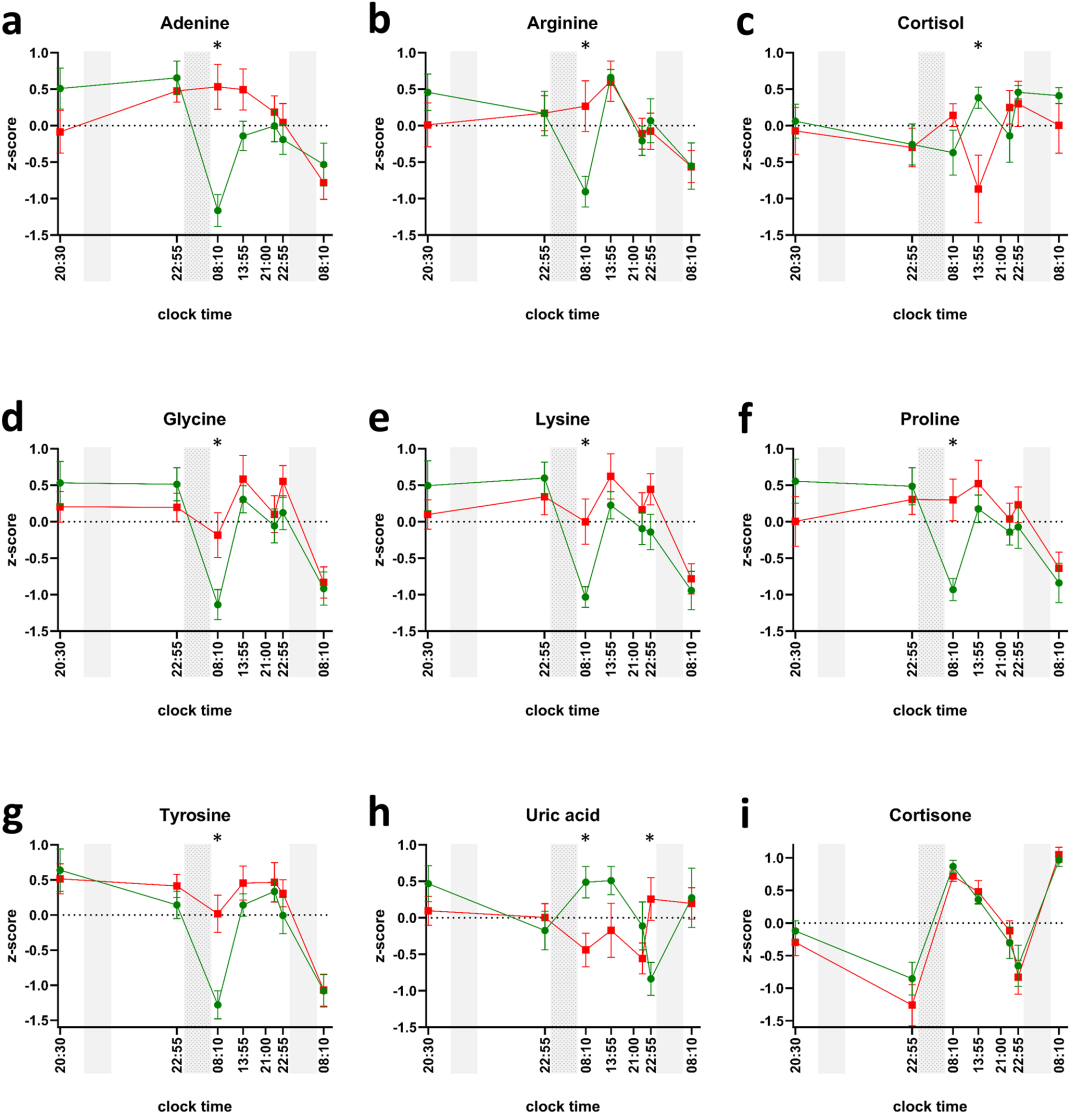
Normalized profiles of significantly changed targeted metabolites (**a**-**h**) and cortisone (**i**). Three gray bars signal biological nighttime (23:30 − 07:30). The second night accentuates that participants in the sleep-deprivation condition were kept awake. Green circles (control condition) and red squares (sleep deprivation condition) indicate mean values of z-scores; whiskers indicate respective standard errors of the mean. Asterisks highlight sampling time points with significant difference between the control and the sleep-deprivation condition (i.e., p-value of paired t-test < 0.05)

Five metabolites (carnitine, propionylcarnitine, butyrylcarnitine, methylmalonic acid, and taurine) did not meet these criteria, as they exhibited significant results at t1 or t2. Six metabolites (adenine, arginine, glycine, lysine, proline, and tyrosine) significantly changed exclusively at t3 (08:10 am, after sleep deprivation), one metabolite (cortisol) exclusively at t4 (01:55 pm), and one metabolite (uric acid) at two time points, namely t3 and t6 (08:10 am and 10:55 pm). The normalized profiles for these eight metabolites are shown in Fig. 4. Naturally, the metabolites found to be significantly altered in the t-test analyses overlap with the ones that were found to be significantly affected by the main effect of ‘sleep’ or the interaction between the two factors ’time’ and ‘sleep’ (see above). For all metabolites that were significantly changed at t3 except uric acid, the measured abundance was higher after sleep deprivation compared to the control condition. In fact, these metabolites are typically low in the early morning hours (at t3 and t7). Sleep deprivation, on the other hand, maintained elevated levels of these metabolites, effectively preventing the occurrence of the morning trough. After the recovery night, however (at t7), both conditions exhibit similar characteristics once more, with a natural morning trough. Lower levels of metabolites for uric acid and cortisol were observed following sleep deprivation in comparison to the control nights at time points t3 and t4, respectively. Out of the 25 targeted metabolites, only uric acid abundance was significantly changed after the maximum period (i.e., after 40 h of sustained wakefulness). However, we observed no evidence of a linear relationship between sustained wakefulness of 15 to 40 h and any changes in single metabolite levels.

For the untargeted metabolomics analysis, 2307 molecular features (RP positive/negative: 695/70, HILIC positive/negative: 1103/439) remained for subsequent statistical analysis after filtering procedures. It is important to mention that the number of authentic metabolites is lower than the number of MF, as metabolites may be duplicated in MF if they are ionizable in both positive and negative mode or show acceptable retention and peak shape characteristics on both RP and HILIC columns. The statistical testing revealed 231 molecular features (10.0% of all detected) with significant alterations in at least one time point. Among these, eight features were found to be significant at two different time points. Considering the different sampling time points, the predominant alterations occurred at t3 (154 MFs, 6.7% of all), with subsequent occurrences observed at t4 (49, 2.1%), t5 (25, 1.1%), and t6 (11, 0.5%). Notably, the majority (76%, 176 out of 231) of the significant MFs were detected in HILIC chromatography. For single analyte results, please refer to Supplementary Information 4.

**Fig. 5:**
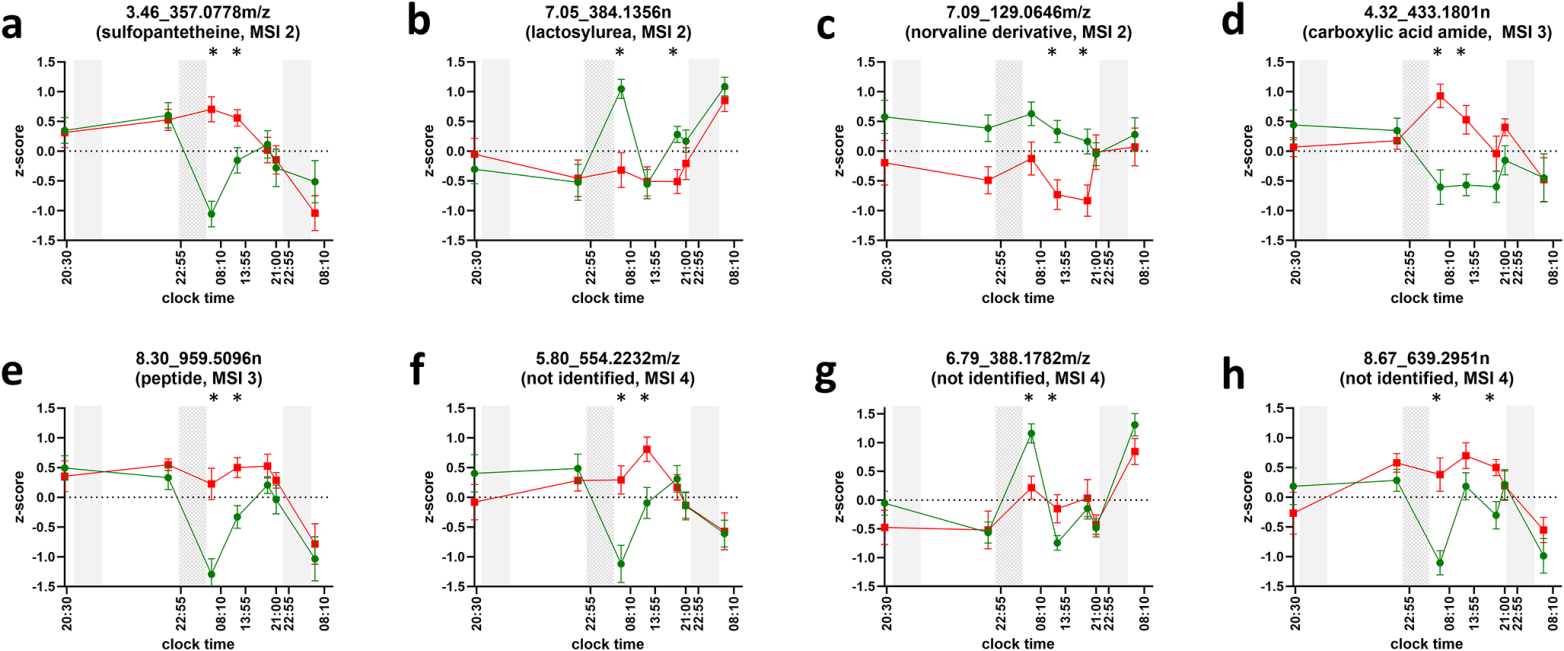
Normalized profiles of untargeted metabolic features with significant changes in two time points. Three gray bars signal biological nighttime (23:30 − 07:30). The second night accentuates that participants in the sleep-deprivation condition were kept awake. Green circles (control condition) and red squares (sleep deprivation condition) indicate mean values of z-scores; whiskers indicate respective standard errors of the mean. Asterisks highlight sampling time points with significant differences between the control and the sleep deprivation condition (i.e., p-value of paired t-test < 0.01). Metabolic features are labeled as retention time, underscore, and mass, the latter being either neutral (n) or m/z. Tentative identifications with MSI levels 2 (a-c), 3 (d,e), 4 (f-h) in brackets underneath, if applicable

In-silico structure annotation analysis of the eight molecular features that were highlighted revealed five different compound classes to be present, namely carboxylic acid amides, peptides, disaccharides, alpha amino acid derivatives, and N-acyl amines (MSI level 3). Furthermore, annotation software could tentatively identify three substances as sulfopantetheine, lactosylurea, and a norvaline derivative (MSI level 2). The normalized profiles of these are displayed in Fig. 5. For their MS2 spectra, please refer to Supplementary Information 5.

## Discussion

In this work, we have investigated the influence of sleep deprivation on metabolite abundances in oral fluid. Monitoring more than 2300 metabolic features employing a semi-targeted metabolomics approach, we found about 10% to have significantly changed in at least one time point after a night of sleep deprivation when compared to 8 h of sleep. The design of the original sleep study allowed us to make use of a pragmatic and hypothesis-driven statistical approach, which allowed us to flag and exclude MF that were either not following adaptive sleep-wake regulation or were of non-robust nature. With additional measures taken regarding significance level adjustment, we made sure that the performed statistical tests were more conservative (i.e., stricter) than conventional limits concerning false positive errors, adding further validity to the detected changes. Consequently, the observed changes are related to the lack of sleep, as they did not occur before sleep deprivation and after recovery sleep. Of these metabolites, eleven could be identified as adenine, arginine, cortisol, glycine, lysine, proline, tyrosine, uric acid, lactosylurea, sulfopantetheine, and a norvaline derivative. Most of the changes could be ascribed to polar substances, as most of the changed metabolic features were detected through HILIC chromatography. The distribution between the two different chromatography columns underlines the prevalent polar properties of the salivary metabolome. This seems understandable as oral fluid consists to its greatest extent of water (Álvarez-Sánchez et al., 2012; Dame et al., 2015). As of practical importance, the majority of metabolites were found to be significantly altered when sampled in the morning hours (here at 08:10 am). Both the results of the targeted and the global metabolomics approach underlined this finding. It was frequently observed in metabolites that exhibited a rhythmical low under control conditions (morning trough) but showed an irregular pattern following sleep deprivation. These observations in oral fluid are consistent with metabolomics analyses of blood plasma samples, which demonstrated that the circadian clock and sleep interact to control the human metabolome (Chua et al., 2015; Davies et al., 2014; Grant et al., 2019). Taken together, these studies showed that up to 20% of all metabolites vary with time of day and that their oscillation can be attenuated or abolished by sleep restriction (Bell et al., 2013) and sleep deprivation (Davies et al., 2014). Particularly amino acids and related biochemicals are typically increased after repeated sleep restriction and sleep deprivation (Bell et al., 2013; Davies et al., 2014). Given the results in blood/plasma and the understanding of salivary secretions as an ultrafiltrate of blood, it seems logical that our findings suggest that the effects of inadequate sleep on the human metabolome can also be detected in oral fluid and may be most pronounced in the morning hours.

We further examined the influence of different sampling time points throughout a day. In numerous metabolomics investigations, the monitoring of participants spans across extended periods of time, encompassing days, weeks, or even months, with the aim of examining the long-term effects of particular stimuli. Consequently, it is frequently observed that the timing of sample collection varies between each visit. In our targeted analysis, 60% of the investigated metabolites were significantly impacted by the factor ‘time’ (time of day), especially if controlled in a certain rhythm. As our study did not apply a constant routine protocol, we were unable to differentiate whether these rhythms were of a circadian or diurnal nature. However, a separate study has reported 15% of the total salivary metabolome to follow circadian paces, with over half of these metabolites being amino acids and their associated compounds (Dallmann et al., 2012). Another study solely focusing on plasma metabolites described about one fifth of these showing significant time-of-day variation (Ang et al., 2012). We found the influence of the factor ‘time’ (i.e., time-of-day variation) most pronounced for the well-described circadian metabolite cortisone and could confirm this phenomenon for the amino acids glycine, arginine, leucine, lysine, ornithine, phenylalanine, tyrosine, and valine. In addition, we could expand this group with nucleobases and derivatives (inosine, uracil, uridine), organic acids (glutaric acid, methylmalonic acid), and butyrylcarnitine. The analysis of metabolites in samples collected at various time points may therefore introduce unintended sources of variation, potentially leading to misinterpretation of the results. We advocate for the implementation of uniform sampling time points in metabolomics studies, particularly in the context of long-term investigations. It should, however, be noted that the described metabolite alterations are not to be understood as potential biomarkers for sleep pressure because each participant’s inner body time (i.e., circadian time) was not monitored. Therefore, we could not directly compare each participant’s sleep pressure at identical circadian times.

Although being widely accepted as an influencing factor, very few metabolomics studies pay attention to the sleep amounts of their participants. The application of monitoring strategies such as actigraphs or sleep/wake diaries is simple and cheap, however. We have shown that a considerable number of metabolites are prone to changes after sleep deprivation, which can lead to subsequent misinterpretations of experimental effects. We therefore encourage all designers of metabolomics studies to ensure a minimum sleep amount and consequent sleep/wake monitoring, e.g., via actigraphy or sleep/wake diaries, irrespective of the study focus. It has to be considered that we have exclusively examined the extreme case of total sleep deprivation (acute sleep deprivation) in this study. The effect of restricted or unsatisfying sleep on the salivary metabolome was not investigated. The study cohort comprised exclusively of male volunteers who were in good health. Consequently, the influence of female gender, age, and health conditions on the observed outcomes could not be assessed. Lastly, the samples used in this study had been stored frozen immediately after sampling for a decade without intermittent thawing. Unfortunately, there are no studies published focusing on the long-term stability of metabolites in oral fluid. However, comparable studies in the more precarious matrices blood and urine stated that time-to-storage, inter-individual differences, and freeze-thaw cycles were by far the strongest factors for variation and instability (Hebels et al., 2013; Stevens et al., 2019). In comparison, storage time and temperature played minor roles, encouraging the use of adequately stored biospecimens, e.g., from biobanks. In this study, all specimens were sampled, stored, and analyzed in the same manner, thus avoiding systematic bias.

## Conclusion

In summary, our study assessed the extent of variation induced by sleep deprivation and different sampling times. These influences were present in metabolites that are frequently monitored in metabolomics studies due to their physiological meaning or association with specific conditions. This may lead to biased result interpretations. We hereby encourage designers of metabolomics studies to mind the following three recommendations: Firstly, apply a within-subject design whenever possible to control for inter-individual variation. Secondly, establish identical time points for sample collection across all participants. Lastly, monitor participants’ sleep-wake schedules prior to and during experiments, even if the study focus is not sleep-related (e.g., via actigraphy).

## Electronic supplementary material

Below is the link to the electronic supplementary material.

Supplementary Material 1

Supplementary Material 2

Supplementary Material 3

Supplementary Material 4

Supplementary Material 5

## Data Availability

The data supporting the findings of this study are available within the paper and its Supplementary Information files. Furthermore, all raw metabolomics data files were uploaded to MetaboLights study identifier MTBLS9176, accessible via www.ebi.ac.uk/metabolights/MTBLS9176.

## Abbreviations

ANOVA: Analysis of variance
DDA: Data-dependent acquisition
ESI: Electrospray ionization
HILIC: Hydrophilic interaction chromatography
HMDB: Human Metabolome Database
HR: High-resolution
ISTD: Internal standard
LC: Liquid chromatography
LOD: Limit of detection
MF: Molecular feature
MS: Mass spectrometry
MSI: Metabolomics Standards Initiative
QC: Quality control
QTOF: Quadrupole-time-of-flight
RP: Reversed-phase (chromatography)
UHPLC: Ultra high performance liquid chromatography
