# Supplementary material for: Assessing the influence of sleep and sampling time on metabolites in oral fluid: implications for metabolomics studies
Source: Metabolomics. 2024 Aug 7;20(5):97. doi: 10.1007/s11306-024-02158-3 (PMC11306311; doi:10.1007/s11306-024-02158-3)
Supplement: Supplementary file 1 — Supplementary Material 1 [file 11306_2024_2158_MOESM1_ESM.docx]

# Supplementary Information 1

## Demographic data of study participants

| n | 13 |
| --- | --- |
| sex | Male (100%) |
| age (years) | 23.2 ± 1.7 (mean ± SD)  22.8 (median) |
| BMI (kg/m^2^) | 22.0 ± 1.9 (mean ± SD)  21.6 (median) |
| alcohol consumption  (drinks/week) | 4.2 ± 3.6 (mean ± SD)  3.5 (median) |
| caffeine consumption*  (mg/day) | 162.6 ± 162.4 (mean ± SD)  120 (median) |

*Caffeine consumption was estimated based on the following average caffeine contents per serving: coffee: 100 mg; Ceylon or green tea: 30 mg; cola drink: 40 mg (2 dL); energy drink: 80 mg (2 dL); chocolate: 50 mg (100 g).
