# Supplementary material for: Assessing the influence of sleep and sampling time on metabolites in oral fluid: implications for metabolomics studies
Source: Metabolomics. 2024 Aug 7;20(5):97. doi: 10.1007/s11306-024-02158-3 (PMC11306311; doi:10.1007/s11306-024-02158-3)
Supplement: Supplementary file 2 — Supplementary Material 2 [file 11306_2024_2158_MOESM2_ESM.docx]

# Supplementary Information 2

## LC-MS analysis setup

LC instrument setup

| Mobile Phase A | 10mM ammonium formate and 0.1 % (v/v) formic acid in water |
| --- | --- |
| Mobile Phase B | 0.1 % (v/v) formic acid in MeOH |
| Mobile Phase C | 25mM ammonium acetate and 0.1% (v/v) acetic acid in water |
| Mobile Phase D | 0.1 % (v/v) acetic acid in ACN |
| Flow rate | 0.5 mL/min |
| RP gradient | 1 minute 100 % A;  1–15 minutes gradual increase to 100 % B;  15–18 minutes held at 100 % B and then decreased to start conditions with flow rate to 0.7 mL/min and re-equilibration for 2 minutes |
| HILIC gradient | 1 minute 5 % C / 95 % D;  1–10 minute gradual change to 60 % C / 40 % D;  10–12‐minute gradual change to 90 % C / 10 % D;  12‐minute hold conditions for 1 minute and then abrupt change to start conditions of 5 % C / 95 % D and re‐equilibration for 4 minutes |
| Column temperature | 40°C |
| Injection volume | positive ionization mode: 1 μL;  negative ionization mode: 5 μL |

MS instrument setup

| Ion source type | IonDrive™ Turbo V at resolving power (full width at half maximum, FWHM at 400 m/z) of 30,000 in MS1 and 15,000 in MS2 (high-sensitivity mode) |
| --- | --- |
| Source temperature | 450°C |
| Curtain gas | 25 psi |
| Ion source gas 1 | 50 psi |
| Ion source gas 2 | 60 psi |
| Ion-spray voltage floating | 5500 V |
| Declustering potential | 80 eV |
| Mass range | m/z 50 to m/z 1000 |
| Accumulation time | 50 msec |
| Collision energy | 35 eV |
| Collision energy spread | 15 eV |
| IDA settings | top 5 MS2, dynamic background subtraction on the five most intense ions; intensity threshold above 100 counts per second (cps); exclusion time of 5 sec (half peak width) after two occurrences |
