# Supplementary material for: Assessing the influence of sleep and sampling time on metabolites in oral fluid: implications for metabolomics studies
Source: Metabolomics. 2024 Aug 7;20(5):97. doi: 10.1007/s11306-024-02158-3 (PMC11306311; doi:10.1007/s11306-024-02158-3)
Supplement: Supplementary file 4 — Supplementary Material 4 [file 11306_2024_2158_MOESM4_ESM.docx]

# Supplementary Information 5

## Analysis 2: Spectra (mgf format) of metabolites that are significantly altered in two time points

BEGIN IONS

PEPMASS=357.077855338568

CHARGE=1-

MSLEVEL=2

Title: Scan#: 1858, RT: 3.4163167 min

80.96532328 48

128.0135161 91

128.0151032 224

128.0166902 94

128.0182773 48

146.0778362 215

146.0795316 283

146.0812269 222

146.0829222 138

146.0846175 82

217.1161612 75

227.014937 48

357.0652236 59

357.0678741 158

357.0705246 304

357.0731752 484

357.0758257 364

357.0784763 610

357.0811268 269

357.0837774 300

357.086428 158

357.0890786 91

357.0917292 48

END IONS

BEGIN IONS

PEPMASS=434.1883

CHARGE=1+

72.07702737 72

72.07940893 71

127.0829033 210

127.0844845 122

127.0860656 92

127.089228 74

127.0908092 68

169.0884681 67

169.090292 131

169.0921158 86

169.0939397 165

169.0957636 210

169.0975874 200

169.0994113 140

169.1012352 209

169.1030591 119

201.0632865 74

201.0652753 84

201.0672642 80

201.069253 153

201.0712419 164

201.0732307 72

306.1253239 74

306.1375942 92

323.1459669 80

323.1535309 97

323.1560523 79

323.1636164 96

323.1661378 92

375.1591839 67

392.1749069 70

416.1741125 97

416.1798352 131

434.170554 126

434.1734766 304

434.1763991 221

434.1793216 310

434.1822442 352

434.1851668 629

434.1880894 1008

434.191012 688

434.1939346 422

434.1968572 319

434.1997798 483

434.2027024 213

434.2056251 69

434.2085477 227

434.2114704 107

434.2173157 72

END IONS

BEGIN IONS

PEPMASS=554.219325902154

CHARGE=1-

Title: Scan#: 3206, RT: 5.7687335 min

277.1099138 36

277.1169188 48

277.1192538 36

554.2063416 76

554.2096437 108

554.2129458 94

554.2162479 101

554.2195501 136

554.2228522 88

554.2261544 193

554.2294566 36

END IONS

BEGIN IONS

PEPMASS=388.1804

CHARGE=1+

73.04504306 193

73.04624178 187

73.04744051 255

73.04863924 106

163.060754 60

250.9655751 60

250.9722409 66

266.9913672 208

266.993659 287

266.9959508 467

266.9982425 166

267.0005343 461

267.0028261 171

267.0051179 163

267.0074098 60

281.0482673 60

355.0617373 169

355.0643801 111

355.067023 79

355.0696659 114

355.0723087 126

355.0749516 110

355.0775945 100

END IONS

BEGIN IONS

PEPMASS=407.1241

CHARGE=1+

245.065037 239

245.0672327 334

245.0694284 380

245.071624 622

245.0738197 473

245.0760154 317

245.0782111 381

245.0804068 148

245.0826026 218

305.0673124 59

305.0722119 136

305.0746617 391

305.0771115 433

305.0795613 146

305.0820111 416

305.0844609 605

305.0869107 570

305.0893605 197

305.0918104 150

305.0942602 105

407.1078382 91

407.1106682 298

407.1134981 262

407.1163281 1173

407.1191581 944

407.121988 1298

407.124818 1815

407.127648 1141

407.1304781 1193

407.1333081 583

407.1361381 657

407.1389682 436

407.1417982 257

407.1446283 223

407.1474584 87

407.1559487 58

END IONS

BEGIN IONS

PEPMASS=129.0643

CHARGE=1+

56.04540493 115

56.04645493 241

56.04750493 589

56.04855495 688

56.04960497 341

56.050655 178

56.05170505 110

56.0527551 60

56.05380516 60

66.03034213 60

66.03262153 217

66.03376124 145

83.05338693 65

83.05466512 245

83.05594332 538

83.05722153 1479

83.05849974 1177

83.05977797 1198

83.06105621 383

83.06233446 311

83.06361272 149

83.06489099 212

83.06616926 130

83.06872585 47

129.0643933 48

END IONS

BEGIN IONS

PEPMASS=960.5161

CHARGE=1+

570.2706014 75

570.2739508 73

570.2773003 89

570.2806497 96

570.2873486 84

570.2906981 123

570.2940476 139

570.3007466 80

570.3040961 99

667.3447543 60

764.3554317 60

764.3670649 60

764.3786982 123

764.382576 98

764.3864538 112

764.3903316 113

764.3942094 157

764.3980873 117

764.4019651 174

764.4058429 96

764.4097208 87

764.4135987 77

764.4174765 93

764.4213544 74

764.4291102 72

764.4329881 60

764.440744 66

960.4606841 82

960.4650309 67

960.4693777 69

960.4737245 114

960.4780713 128

960.4824182 137

960.486765 121

960.4911119 274

960.4954587 214

960.4998056 394

960.5041525 348

960.5084994 416

960.5128463 342

960.5171932 366

960.5215401 362

960.5258871 487

960.530234 352

960.534581 171

960.5389279 245

960.5432749 98

960.5476219 137

960.5519689 118

960.5563159 224

960.5606629 120

960.5650099 108

960.5693569 75

960.573704 103

END IONS

BEGIN IONS

PEPMASS=640.3033

CHARGE=1+

583 45

640 704.75

END IONS
